# The genus *Omalus* Panzer, 1801 (Hymenoptera, Chrysididae) from China, with descriptions of four new species

**DOI:** 10.3897/zookeys.407.7531

**Published:** 2014-05-08

**Authors:** Na-sen Wei, Paolo Rosa, Jing-xian Liu, Zai-fu Xu

**Affiliations:** 1 Department of Entomology, College of Natural Resources and Environment, South China Agricultural University, Guangzhou 510640, P. R. China; 2 Via Belvedere 8/d, I-20881 Bernareggio (MB), Italy

**Keywords:** Chrysididae, *Omalus*, new species, Palaearctic, Oriental, China

## Abstract

The Chinese species of the genus *Omalus* Panzer, 1801 are revised and keyed for the first time. Eight species are recorded, of which four are new to science and one is new to China: *Omalus aeneus* (Fabricius, 1787), *Omalus berezovskii* (Semenov-Tian-Shanskij, 1932), *Omalus potanini* (Semenov-Tian-Shanskij, 1932), *Omalus imbecillus* (Mocsáry, 1889) (new to China), *Omalus helanshanus* **sp. n.**, *Omalus probiaccinctus* **sp. n.**, *Omalus pseudoimbecillus* **sp. n.**, and *Omalus tibetanus* **sp. n.**

## Introduction

The genus *Omalus* Panzer, 1801 belongs to the chrysidid tribe Elampini (Chrysidinae). This genus is differently interpreted by different authors, and the complex history of this genus was summarised by Rosa (2006). In this study, we follow the system used for Fauna Europaea (Rosa and Soon 2013), which is slightly altered according to the system of Kimsey and Bohart (1991).

Currently *Omalus* includes 26 species (Kimsey and Bohart 1991; Rosa 2005, 2006, 2009). In China, only three species of *Omalus* were known before this study, *Omalus aeneus* (Fabricius, 1787), *Omalus berezovskii* (Semenov-Tian-Shanskij, 1932) and *Omalus potanini* (Semenov-Tian-Shanskij, 1932). In this paper, four new species of *Omalus*, *Omalus helanshanus* sp. n., *Omalus probiaccinctus* sp. n., *Omalus pseudoimbecillus* sp. n., and *Omalus tibetanus* sp. n., and one new to China, *Omalus imbecillus* (Mocsáry, 1889) are described, and a key to all eight species is also provided.

## Materials and methods

All specimens were examined and described under a stereomicroscope (Olympus SZ61). All photos were taken with a digital camera (CoolSNAP) attached to a Zeiss Stemi 2000-CS stereomicroscope. Images were processed using Image-Pro Plus software.

Morphological terminology follows Kimsey and Bohart (1991). Abbreviations used in the descriptions are as follows: F-I, F-II, F-III, etc. = flagellum I, flagellum II, flagellum III and so on; L/W = relative length to width; MOD = midocellar diameter; MS = malar space, the shortest distance between the base of the mandible and the margin of the compound eyes; Notaulic pit = the pit on the posterior margin of mesoscutum where notauli originate; PD = puncture diameter; T-I, T-II, T-III, etc. = metasomal tergum I, tergum II, tergum III and so on.

Types and other specimens have been examined from the following institutions:

HNHM Hungarian Natural History Museum, Budapest, Hungary.

SCAU Hymenopteran Collection, South China Agricultural University, Guangzhou, China.

SHEM Shanghai Entomological Museum, Chinese Academy of Sciences, Shanghai, China.

ZMUC Zoological Museum, University of Copenhagen, Copenhagen, Denmark.

## Systematics

### Genus Omalus

Panzer, 1801

http://species-id.net/wiki/Omalus

Omalus Panzer, 1801: 13. Type species: *Chrysis aenea* Fabricius, 1787. Bohart and Campos 1960: 235 (partim, *Omalus* s. s.); Bohart and Kimsey 1982: 36 (partim, *Omalus* s. s.); Kimsey and Bohart 1991: 243; Rosa 2005: 8; 2006: 100.

#### Diagnosis.

This genus is close to *Holophris* Mocsary, 1890, *Philoctetes* Abeille, 1879, and *Pseudomalus* Ashmead, 1902, but can be distinguish by mesoscutum impunctate and transpleural carina reaching the apex of propodeal angle. Other diagnostic characteristics are: scapal basin deep, smooth and glabrous, rarely with weak striae; malar space equal to or longer than 1 MOD, rarely less than 1 MOD, and horizontally bisected by the genal carina; mandibles tridentate; pronotum impunctate medially or nearly so; mesopleuron with scrobal sulcus horizontally, and with a single carina dorsally; transpleural carina reaching the apex of propodeal angle; scutellum with two flattened foveae on anterior margin; metanotum round or hemispherical; tarsal claw with three to six teeth; apex of T-III usually with small medial notch, rarely absent.

#### Biology.

Species of *Omalus* have been reported as parasitoids of some crabronid wasps (Mocsáry 1889; Tsuneki 1952; Krombein 1963, 1967; Parker and Bohart 1966; Nozaka 1969; Bohart and Kimsey 1982; Tormos et al. 1996; Rosa 2006).

#### Distribution.

*Omalus* occurs in all zoogeographic regions, except Australia. There are 26 valid *Omalus* species, of which 19 are found in the Palaearctic, one in both the Holarctic and the Oriental, three in the Nearctic, two in the Afrotropical, and one in the Neotropical Regions.

#### Key to the Chinese species of the genus *Omalus* Panzer

**Table:** 

1	Tarsal claw with four teeth	2
–	Tarsal claw with three teeth	3
2	Scapal basin with weak, transverse striae (Plate 12A); mesoscutum transversally rugulose, with notauli distinct, deep and complete (Plate 12D); metasoma distinctly elongate (Plate 12E); apex of T-III with faint median notch (Plate 12F)	*Omalus probiaccinctus* sp. n.
–	Scapal basin smooth, without transverse striae (Plates 2A, 4A); mesoscutum polished, with notauli indistinct but complete, impressed as fine lines (Plates 2D, 4D); metasoma oval (Plates 2E, 4E); apex of T-III with distinct median notch (Plates 2F, 4F)	*Omalus aeneus* (Fabricius)
3	Notauli distinct and deep (Plates 6D, 16D)	4
–	Notauli indistinct, impressed as fine lines (Plates 8D, 10D, 14D)	5
4	Pronotum with scattered (1–2 PD), shallow punctures medially (Plate 6B); metasoma pear-shaped, T-II notably wider than T-I; apex of T-III with median notch V-shaped (Plate 6F)	*Omalus berezovskii* (Semenov-Tian-Shanskij)
–	Pronotum almost impunctate medially (Plate 16B); metasoma distinctly elongate, T-II not wider than T-I; apex of T-III with median notch shallowly indented (Plate 16F)	*Omalus tibetanus* sp. n.
5	Scutellum with two flattened and semi-elliptical foveae on anterior margin (Plates 10D, 14D)	6
–	Scutellum without foveae on anterior margin (Plate 8D)	7
6	Tegula fully metallic blue (Plate 10B); mesopleuron without striae between punctures (Plate 10C); propodeal angle indistinct	*Omalus imbecillus* (Mocsáry)
–	Tegula transparent brownish, with faint metallic reflections anteriorly (Plate 14B); mesopleuron with striae between punctures (Plate 14C); propodeal angle distinct and stout	*Omalus pseudoimbecillus* sp. n.
7	Body metallic bluish-purple, with green reflections; apex of T-III with brownish transparent rim (Plate 8F)	*Omalus helanshanus* sp. n.
–	Body metallic green-bronzy, with more or less golden-green reflections; apex of T-III with colourless transparent rim	*Omalus potanini* (Semenov-Tian-Shanskij)

### Omalus aeneus

(Fabricius, 1787)

http://species-id.net/wiki/Omalus_aeneus

**Plate 1.**
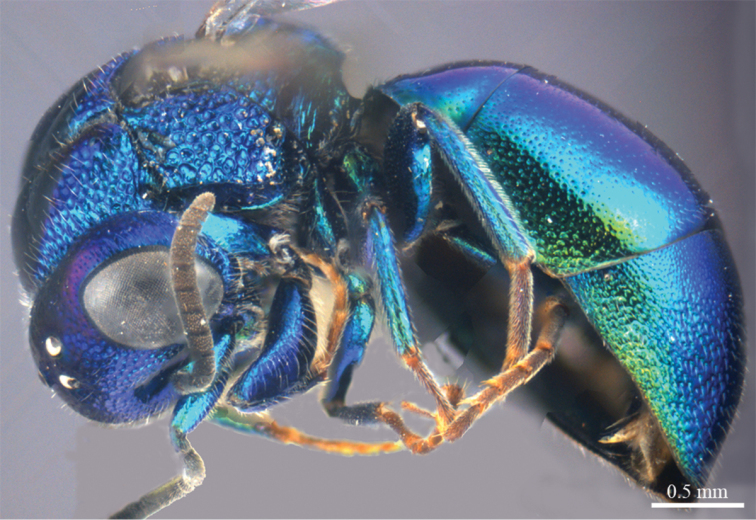
*Omalus aeneus* (Fabricius, 1787), female from Inner Mongolia. Habitus lateral.

Plates 1 [Fig F2] [Fig F3] –4

Chrysis aenea Fabricius, 1787: 284.
Omalus aeneus (Fabricius, 1787): Panzer 1801: 13; Kimsey and Bohart 1991: 245; Kunz 1994: 74; Mingo 1994: 78; Rosa 2006: 101.
Elampus chevrieri Tournier, 1877: 105 (synonymized by Kimsey and Bohart 1991).
Omalus aeneus var. *pygialis* du Buysson, 1887: 170 (synonymized by Kimsey and Bohart 1991).
Philoctetes japonicus Bischoff, 1910: 438 (synonymized by Kimsey and Bohart 1991).
Ellampus sauteri Mocsáry, 1913: 613 (synonymized by Kimsey and Bohart 1991).

#### Material examined.

Type material: 1 ♀ (ZMUC), *aenea* [handwritten by Fabricius] [specimen considered as Type by Zimsen (1964) and Kimsey and Bohart (1991)]; 1 ♀ (HNHM), “Formosa Sauter”, “Taihorinsho, 1909. XI.”, “sauteri Mocs. type, det. Mocsáry”, “Holotypus Ellampus sauteri, ♀, Mocsáry, (L. D. French)”, “id nr. 134845, HNHM, Hym. coll.”. Other material: 1 ♀ (SCAU), Inner Mongolia, Helanshan, Gulaben, Dayanggou (39°5'24.90"N, 106°3'32.35"E), 27.VII.2010, Hong-fei Chai, No. SCAU-O0001; 2 ♀♀ (SCAU), Inner Mongolia, Helanshan, Halawuchagou (38°51'33.33"N, 105°53'28.67"E), 10.VIII.2010, Hong-fei Chai, No. SCAU-O0002, SCAU-O0003; 2 ♀♀ (SCAU), Inner Mongolia, Helanshan, Shuimogou (38°57'25.97"N, 105°52'22.90"E), 30.VII.2010, Jie Zeng, No. SCAU-O0004, SCAU-O0005; 1 ♂ (SCAU), Inner Mongolia, Helanshan, Shuimogou, 30.VII.2010, Jie Zeng, No. SCAU-O0006; 1 ♂ (SCAU), Inner Mongolia, Helanshan, Qianggangling (38°53'N, 105°59'E ), 3.VIII.2010, Jie Zeng, No. SCAU-O0007; 1 ♂ (SCAU), Inner Mongolia, Helanshan, Halawuchagou 10.VIII.2010, Hong-fei Chai, No. SCAU-O0008; 1 ♀ (SHEM), Inner Mongolia, Helanshan, Gulaben (38°53'N, 105°59'E ), 2700 m, 27.VIII.2010, Xu-feng Zhang and Feng-li Cui, No. 34020542.

#### Diagnosis.

Scapal basin deep, smooth and glabrous. Mesoscutum polished, with notauli indistinct but complete, impressed as fine lines. Propodeum with lateral margin distinctly concave before propodeal angle. Tarsal claw with four teeth. Metasoma oval; apex of T-III with distinct median notch.

#### Description.

*Female* (n = 12). Body length 4.5–5.4 mm (Plate 1). Fore wing length 3.3–4.2 mm. MS = 0.9 MOD.

**Plate 2. F2:**
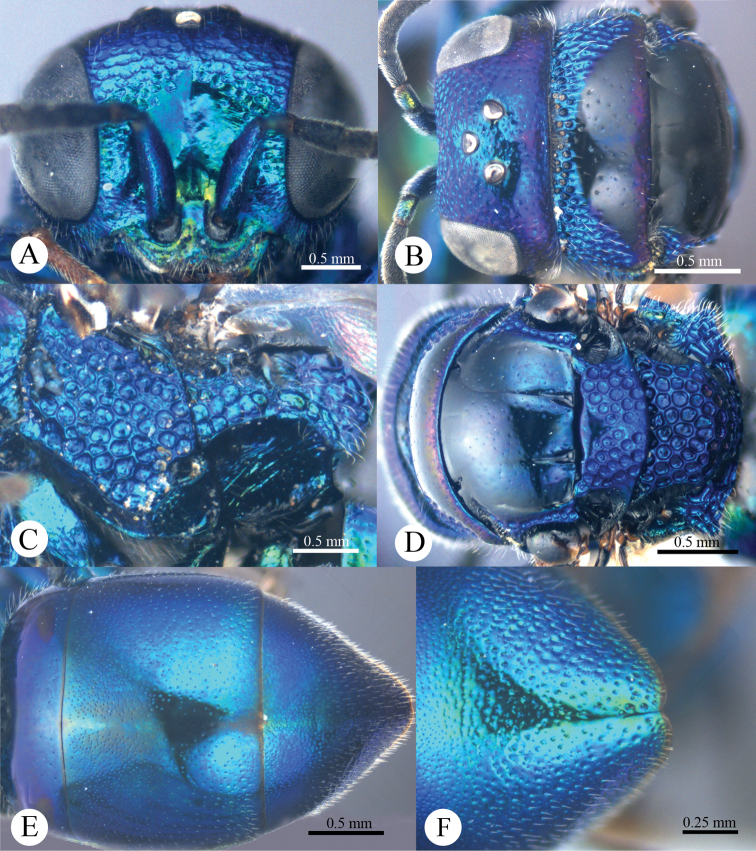
*Omalus aeneus* (Fabricius, 1787), female from Inner Mongolia. **A** Head anterior **B** Head and pronotum dorsal **C** Mesopleuron and metapleuron lateral **D** Mesoscutum, scutellum, metanotum and propodeum dorsal **E** Metasoma dorsal **F** Apex of T-III dorsal.

*Head*. Face with large, round, dense (0–0.5 PD), shallow punctures (Plate 2A). Scapal basin deep, smooth and glabrous (Plate 2A). Ocellar triangle isosceles. Postocellar line absent (Plate 2B). Gena with fine and oblique wrinkles.

*Mesosoma*. Pronotum almost impunctate medially, with small, deep pits on anterior margin; with large, dense (0–0.5 PD) punctures laterally and anteriorly towards the collar (Plate 2B). Mesoscutum polished, almost impunctate (Plate 2D); notauli indistinct but complete, impressed as fine lines; notaulic pit elongate; parapsidal lines indistinct, similar to notauli (Plate 2D). Scutellum without flattened fovea on anterior margin; with small, triangular and impunctate area antero-medially, with deep, round, dense (0–0.5 PD) punctures, and becoming larger towards alar foveae (Plate 2D). Mesopleuron without striae between punctures (Plate 2C). Metanotum evenly round, with large, deep, areolate-reticulate punctures (Plate 2D). Propodeum with lateral margin distinctly concave before propodeal angle (Plate 2D); propodeal angle distinct and stout, pointing posterolaterally (Plate 2D). Tarsal claw with four teeth.

*Metasoma*. Oval (Plate 2E), L/W = 11/7. T-I almost impunctate. T-II with fine, dense punctures. T-III with fine, much denser punctures than those on T-II (Plate 2E); apex of T-III with narrow (1/3 MOD), brownish transparent rim, with distinct median notch (Plate 2F).

*Colouration*. Face metallic blue. Vertex and mesosoma metallic bluish-purple, with medial pronotum and mesoscutum blackish. Antenna black, with scape and pedicel metallic green. Tegula blackish-brown. Leg metallic bluish-green, with tarsus brown. Metasoma metallic greenish-blue, with blackish tints.

**Plate 3. F3:**
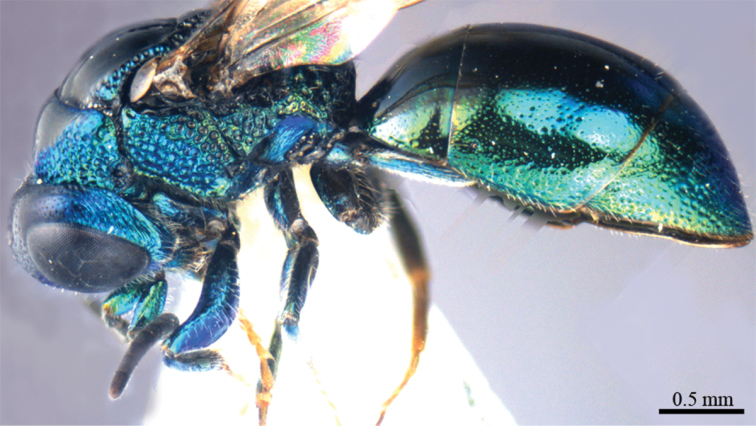
*Omalus aeneus* (Fabricius, 1787), male from Inner Mongolia. Habitus lateral.

**Plate 4.**
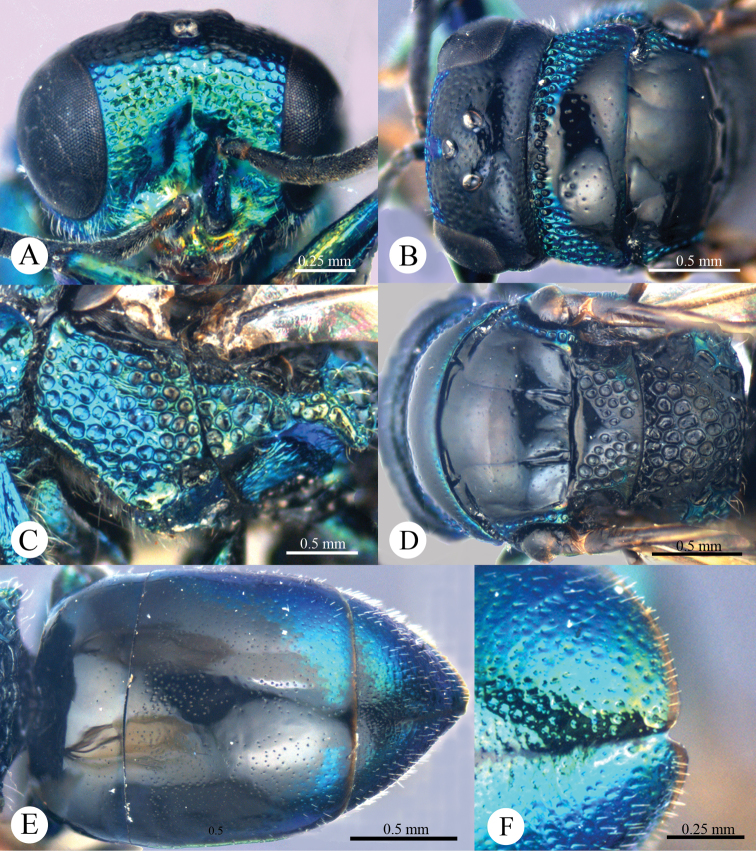
*Omalus aeneus* (Fabricius, 1787), male from Inner Mongolia. **A** Head anterior **B** Head and pronotum dorsal **C** Mesopleuron and metapleuron lateral **D** Mesoscutum, scutellum, metanotum and propodeum dorsal **E** Metasoma dorsal **F** Apex of T-III dorsal.

*Male* (n = 3). Body length 4.4–5.0 mm (Plate 3). Forewing length 3.2–3.9 mm. POL: OOL: OCL = 6.2: 6.4: 6.0. MS = 1.0 MOD. Differing from female as follows: face metallic greenish blue; vertex, medial pronotum, mesonotum, metanotum and propodeum blackish (Plate 4B, 4D); with antero-lateral corners of pronotum, mesopleuron, metapleuron, and lateral part of propodeum metallic green; metasoma blackish with metallic green on posterior T-II and T-III (Plate 4E); apex of T-III with median notch deeper than that of female (Plate 4F).

#### Distribution.

China (Inner Mongolia, Taiwan); Japan; widespread in Holarctic (Kimsey and Bohart 1991; Kunz 1994; Mingo 1994; Rosa 2006).

#### Biology.

Collected from June to November (Tsuneki 1970; Rosa 2006). Two or more generations are observed in South Europe in one year (Rosa 2006). Hosts include species in the genera *Pemphredon*, *Passaloecus* and *Psenulus* (Crabronidae) (Mocsáry 1889; Grandi 1961; Kunz 1994; Strumia 1997).

### Omalus berezovskii

(Semenov-Tian-Shanskij, 1932)

http://species-id.net/wiki/Omalus_berezovskii

Plates 5 , 6

Ellampus berezovskii Semenov-Tian-Shanskij, 1932: 12.
Omalus berezovskii (Semenov-Tian-Shanskij, 1932): Kimsey and Bohart 1991: 247.

#### Material examined.

2 ♀♀ (SCAU), Ningxia, Liupanshan Forest Park (35°22'54.76"N, 106°18'51.22"E), 3–4.VII.2009, Hua-yan Chen, No. SCAU-O0009, SCAU-O0010.

#### Diagnosis.

Pronotum with scattered (1–2 PD), shallow punctures medially. Mesoscutum with notauli distinct, deep, complete. Mesopleuron with distinct striae between punctures. Metasoma pear-shaped, T-II notably wider than T-I. Apex of T-III with median notch V-shaped.

#### Description.

**Plate 5.**
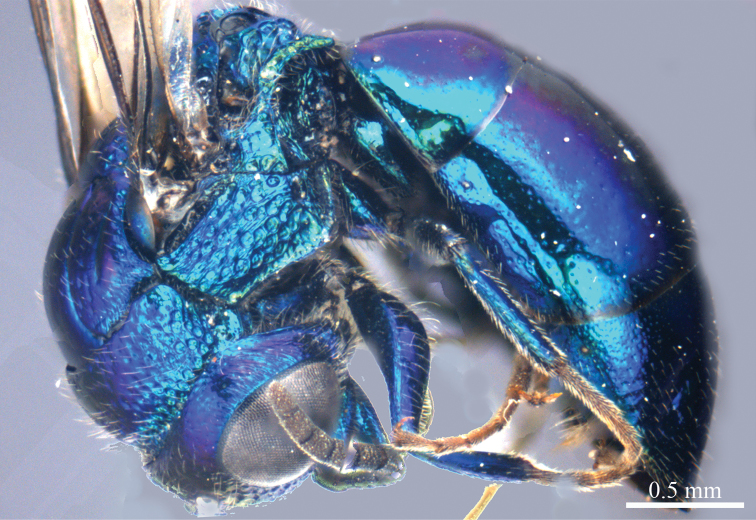
*Omalus berezovskii* (Semenov-Tian-Shanskij, 1932), female from Ningxia. Habitus lateral.

*Female* (n = 2). Body length 4.1–4.6 mm (Plate 5). Fore wing length 3.3–4.0 mm. MS = 1.7 MOD.

**Plate 6.**
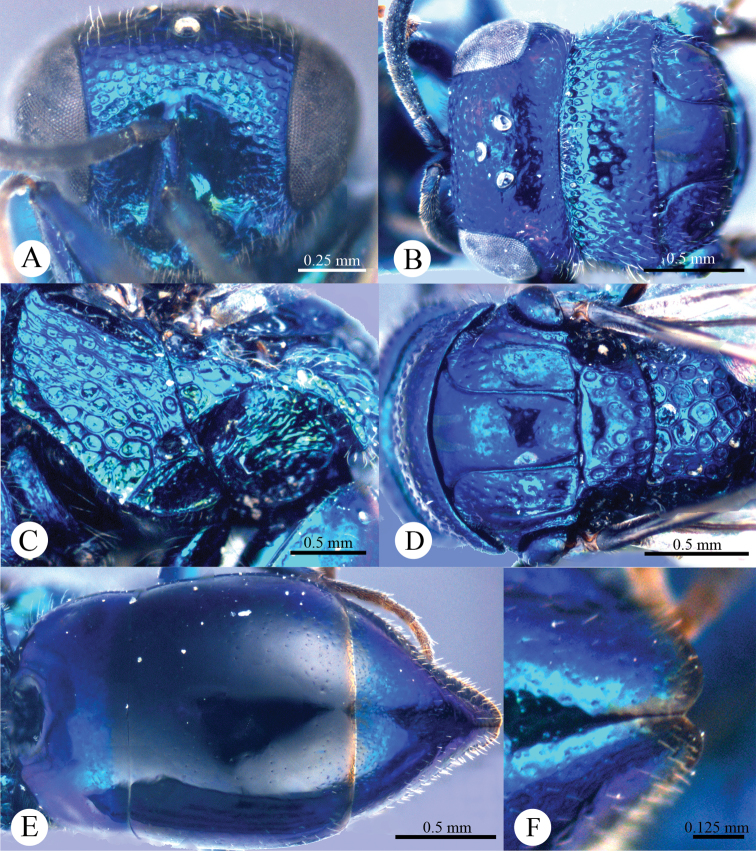
*Omalus berezovskii* (Semenov-Tian-Shanskij, 1932), female from Ningxia. **A** Head anterior **B** Head and pronotum dorsal **C** Mesopleuron and metapleuron lateral **D** Mesoscutum, scutellum, metanotum and propodeum dorsal **E** Metasoma dorsal **F** Apex of T-III dorsal.

*Head*. Face with round, dense (0–0.5 PD), shallow punctures (Plate 6A). Scapal basin deep, smooth, glabrous, weakly striae laterally near antennal sockets (Plate 6A). Ocellar triangle isosceles. Postocellar line absent (Plate 6B). Gena with fine, transverse wrinkles.

*Mesosoma*. Pronotum with scattered (1–2 PD), shallow punctures medially (Plate 6B); with small, deep pits on anterior margin; with large, dense (0–0.5 PD) punctures laterally (Plate 6B). Mesoscutum polished, with fine, scattered punctures between notauli; with dense, deep punctures between parapsidal lines and notauli (Plate 6D); notauli distinct, deep, complete; notaulic pit elongate; parapsidal lines shallower than notauli (Plate 6D). Scutellum without flattened fovea on anterior margin; with triangular and impunctate area antero-medially (Plate 6D); with shallow punctures laterally, and becoming larger and deeper towards alar foveae (Plate 6D). Mesopleuron with distinct striae between the punctures (Plate 6C). Metanotum evenly round, with large, deep, areolate punctures (Plate 6D). Propodeum with lateral margin slightly concave before propodeal angle; propodeal angle distinct, long, pointing posterolaterally. Tarsal claw with three teeth.

*Metasoma*. Oval, T-II notably wider than T-I, T-III distinctly constrict laterally towards the apex (Plate 6E), L/W = 9/5. T-I and T-II almost impunctate dorsally, with fine, scattered punctures laterally. T-III with finer, denser punctures than those on T-II (Plate 6E); apex of T-III with narrow (2/3 MOD) and brownish rim, with median notch V-shaped, 1/2 MOD deep (Plate 6F).

*Colouration*. Head and mesosoma mostly metallic bluish-purple, with face, mesopleuron and metapleuron metallic green. Antenna black, with scapes and pedicel metallic greenish-blue. Tegula metallic green, with apex brown. Leg metallic bluish-purple, with tarsus brown. Metasoma metallic blue with purple reflections, with T-II blackish dorsally.

*Male*. Unknown.

#### Distribution.

China (Ningxia, Sichuan) (Semenov-Tian-Shanskij 1932).

#### Biology.

Unknown. Collected from May to July (Semenov-Tian-Shanskij 1932; Kimsey and Bohart 1991).

### Omalus helanshanus sp. n.

http://zoobank.org/B6E18E24-218C-41F2-A6ED-028CA872C119

http://species-id.net/wiki/Omalus_helanshanus

Plates 7 , 8

#### Material examined.

Holotype, ♀ (SCAU), Inner Mongolia, Helanshan, Gulaben, Dayanggou (39°5'24.90"N, 106°3'32.35"E), 27.VII.2010, Hong-fei Chai, No. SCAU-O0011. Paratypes: 3 ♀♀ (SCAU), Inner Mongolia, Helanshan, Gulaben, Dayanggou, 27.VII.2010, Hong-fei Chai, No. SCAU-O0012–0014; 2 ♀♀ (SCAU), Inner Mongolia, Helanshan, Shuimogou (38°57'25.97"N, 105°52'22.90"E), 30.VII.2010, Hong-fei Chai, No. SCAU-O0015, SCAU-O0016; 1 ♀ (SCAU), Inner Mongolia, Helanshan, Shuimogou, 30.VII.2010, Jie Zeng, No. SCAU-O0017; 3 ♀♀ (SCAU), Inner Mongolia, Helanshan, Habeigou, Huangliangzi (38°51'49.22"N, 105°53'17.40"E), 9.VIII.2010, Jie Zeng, No. SCAU-O0018–O0020; 5 ♀♀ (SCAU), Inner Mongolia, Helanshan, Halawuchagou (38°51'40.66"N, 105°52'10.49"E), 10.VIII.2010, Hong-fei Chai, No. SCAU-O0021–O0025; 1 ♀ (SCAU), Inner Mongolia, Helanshan, Halawuchagou, 10.VIII.2010, Cheng-jin Yan, No. SCAU-O0026; 11 ♀♀ (SCAU), Inner Mongolia, Helanshan, Halawubeigou (38°51'33.33"N, 105°53'28.67"E), 9.VIII.2010, Hong-fei Chai, No. SCAU-O0027–0037; 1 ♀ (SHEM), Inner Mongolia, Helanshan, Halawu (38°52'5.39"N, 105°45'29.34"E), 2250 m, 10.VIII.2010, Xu-feng Zhang and Feng-li Cui, No. 34020573; 1 ♀ (SHEM), Inner Mongolia, Helanshan, Halawu, 2800 m, 9.VIII.2010, Xu-feng Zhang and Feng-li Cui, No. 34020592; 1 ♀ (SHEM), Inner Mongolia, Helanshan, Halawu, 2250 m, 10.VIII.2010, Xu-feng Zhang and Feng-li Cui, No. 34020129.

#### Diagnosis.

*Omalus helanshanus* sp. n. is similar to *Omalus potanini* Semenov-Tian-Shanskij based on the indistinct notauli and scutellum without flattened fovea on anterior margin; it also resembles *Omalus tibetanus* sp. n. based on the elongate metasoma and the similar sculpture. However, *Omalus helanshanus* sp. n. can be distinguished by the combination of the following characters: postocellar line present, apex of T-III with brownish transparent rim and median notch faint. Comparison with *Omalus potanini* Semenov-Tian-Shanskij is based on the description of the male, since the female of *Omalus potanini* is unknown.

#### Description.

**Plate 7.**
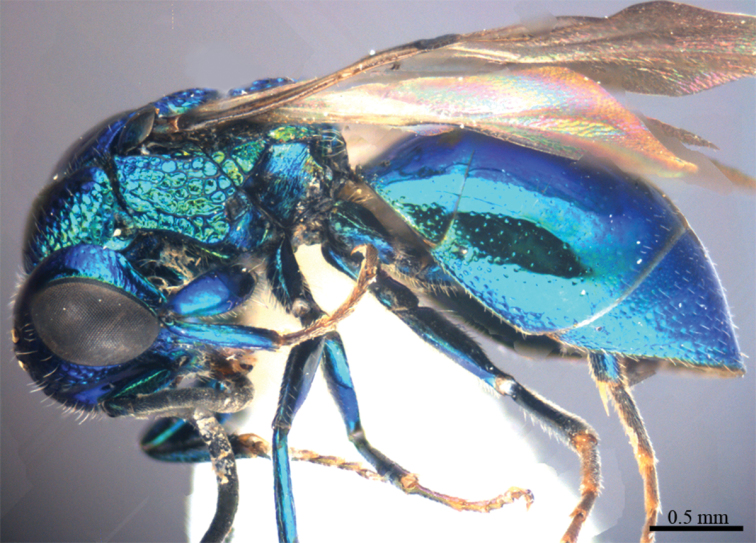
*Omalus helanshanus* sp. n., holotype, female. Habitus lateral.

*Female* (n = 30). Body length 3.9–4.9 mm (Plate 7). Forewing length 3.1–4.4 mm. MS = 0.9 MOD.

**Plate 8.**
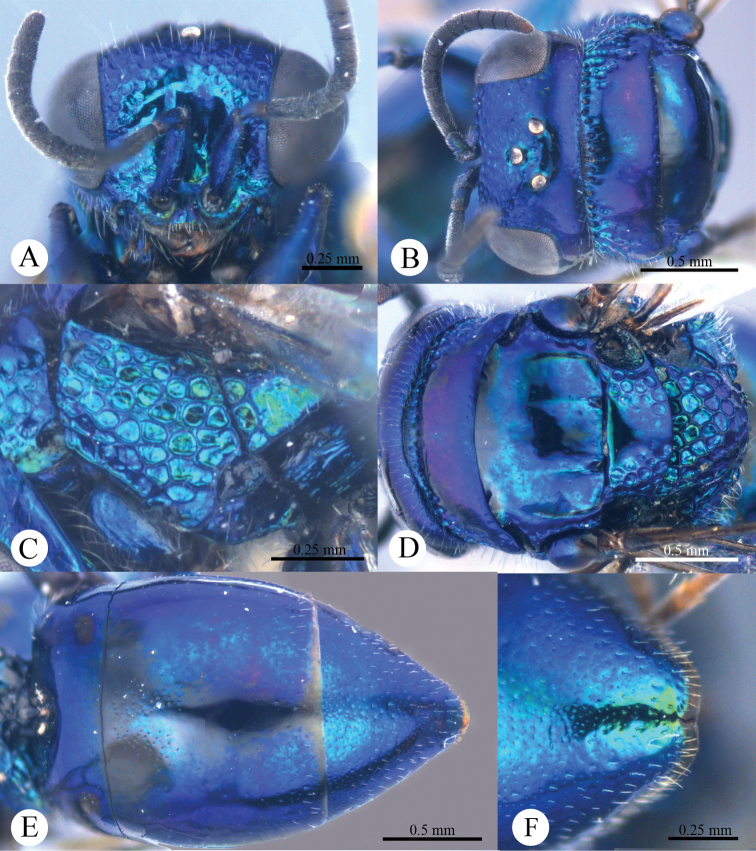
*Omalus helanshanus* sp. n., holotype, female. **A** Head anterior **B** Head and pronotum dorsal **C** Mesopleuron and metapleuron lateral **D** Mesoscutum, scutellum, metanotum and propodeum dorsal **E** Metasoma dorsal **F** Apex of T-III dorsal.

*Head*. Face with large, round, dense (0–0.5 PD), shallow punctures (Plate 8A). Scapal basin deep, smooth, glabrous (Plate 8A). Ocellar triangle isosceles. Postocellar line shallowly impressed (Plate 8B). Gena without wrinkles or with very fine wrinkles.

*Mesosoma*. Pronotum almost impunctate medially, with small, deep pits on anterior margin; with large, dense (0–0.5 PD) punctures laterally (Plate 8B). Mesoscutum polished, almost impunctate (Plate 8D); notauli indistinct, almost complete, impressed as fine lines, with notaulic pit round and short; parapsidal lines indistinct (Plate 8D). Scutellum without flattened fovea on anterior margin; with broad, impunctate median area extending along its length, slightly convergent posteriorly (Plate 8D); with shallow, areolate punctures laterally, becoming larger and deeper towards alar foveae. Mesopleuron without striae between punctures (Plate 8C). Metanotum evenly round, with large, deep, areolate-reticulate punctation (Plate 8D). Propodeum with lateral margin concave before propodeal angle; propodeal angle small, short, pointing backwards (Plate 8D). Tarsal claw with three teeth.

*Metasoma*. Elongate (Plate 8E), L/W = 10/7. T-I and T-II almost impunctate dorsally, with fine, scattered punctures towards the margins and laterally (Plate 8E). T-III with fine, denser punctures than those on T-II; apex of T-III with narrow (1/2 MOD), brownish transparent rim, with faint median notch (Plate 8F).

*Colouration*. Head and mesosoma mostly metallic bluish-purple, with distinct or faint metallic green reflections on mesopleuron, metanotum, metapleuron, and propodeum. Antenna black, with scape and pedicel metallic green. Tegula purple, with apex brownish. Leg metallic bluish-purple, with tarsus brown. Metasoma metallic bluish-purple, with some metallic green reflections.

*Male*. Unknown.

#### Distribution.

China (Inner Mongolia).

#### Biology.

Unknown. Collected from July to August.

#### Etymology.

The species is named after the type locality.

### Omalus imbecillus

(Mocsáry, 1889) (new to China)

http://species-id.net/wiki/Omalus_imbecillus

Plates 9 , 10

Ellampus imbecillus Mocsáry, 1889: 98.
Holophris imbecillus (Mocsáry, 1889): Kimsey and Bohart 1991: 225.
Omalus imbecillus (Mocsáry, 1889): Rosa 2005: 12.

#### Material examined.

Lectotype: 1 ♀ (HNHM), “Turkestan”, “*imbecillus* Mocs, type, det. Mocsáry”, “Lectotypus, *Ellampus imbecillus*, ♀, (L. D. French), Mocsáry”, “id nr. 135046, HNHM, Hym. coll.”. Other material: 3 ♀♀ (HNHM), Laos, Vientiane, 19.III.1990, E. Kondorosy; 1 ♀ (SCAU), Yunnan, Kaiyuannan River (23°42'N, 103°16'E ), 16.VII.2003, Long Hu, No. 20048184; 1 ♀ (SCAU), Yunnan, Gaoligongshan National Nature Reserve (25°50'23"N, 98°51'23"E), 16–17.VII.2006, Jie Zeng, Juan-juan Ma, and Bin Xiao, No. SCAU-O0038.

#### Diagnosis.

Scutellum with two flattened and semi-elliptical foveae on anterior margin, with numerous fine, short, longitudinal striae on posterior margin. Propodeal angle indistinct. Apex of T-III with narrow, colourless semi-transparent rim, without medial notch.

#### Description.

**Plate 9.**
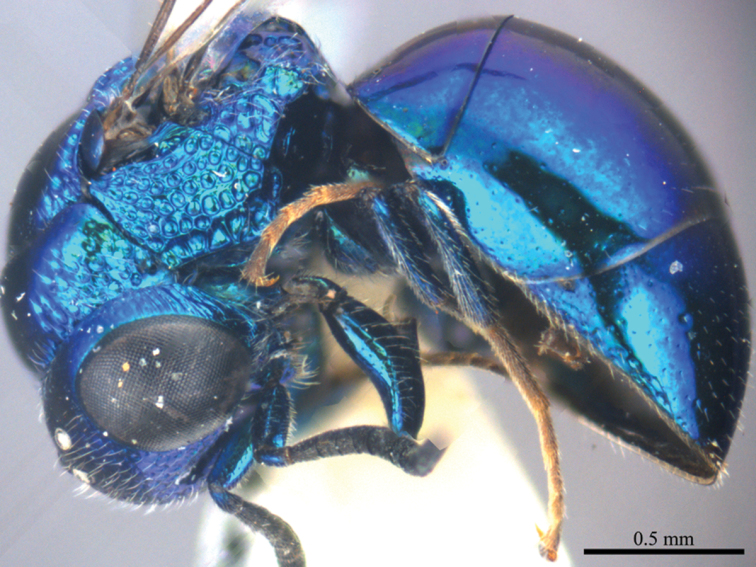
*Omalus imbecillus* (Mocsáry, 1889), female from Yunnan. Habitus lateral.

Described from a female from Yunnan. Body length 3.3 mm (Plate 9). Forewing length 2.6 mm. MS = 1.0 MOD.

**Plate 10.**
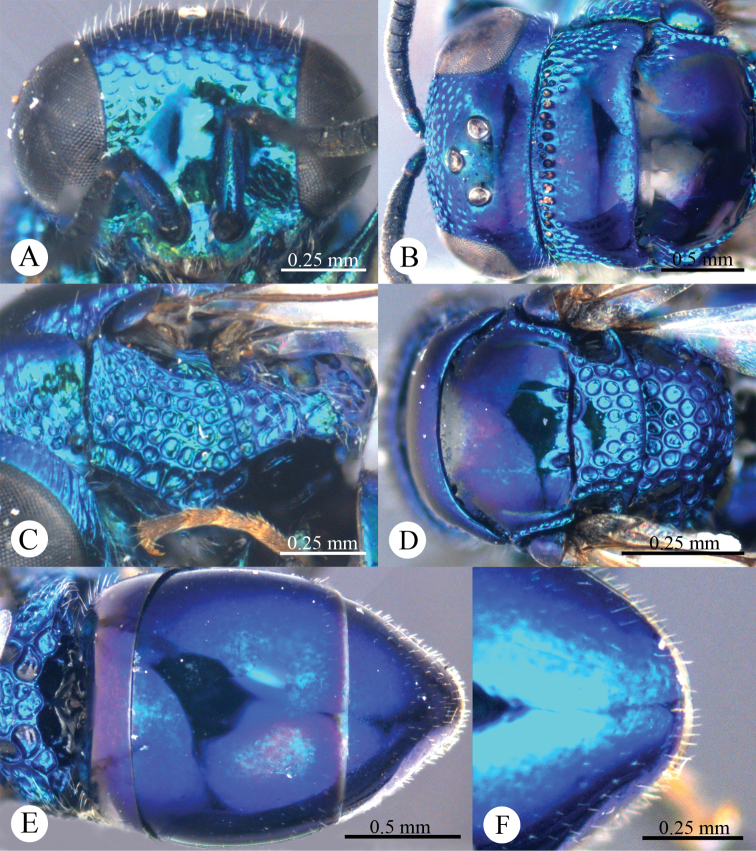
*Omalus imbecillus* (Mocsáry, 1889), female from Yunnan. **A** Head anterior **B** Head and pronotum dorsal **C** Mesopleuron and metapleuron lateral **D** Mesoscutum, scutellum, metanotum and propodeum dorsal **E** Metasoma dorsal **F** Apex of T-III dorsal.

*Head*. Face with large, round, dense (0–0.5 PD), shallow punctures (Plate 10A). Scapal basin deep, smooth, glabrous (Plate 10A). Ocellar triangle isosceles. Postocellar line very weak, broadly interrupted medially (Plate 10B). Gena with distinct, oblique wrinkles.

*Mesosoma*. Pronotum impunctate medially, with small, deep pits on anterior margin; with large, dense (0–0.5 PD) punctures laterally (Plate 10B). Mesoscutum polished, impunctate (Plate 10D); notauli indistinct but complete, impressed as fine lines, with notaulic pit oval; parapsidal lines indistinct (Plate 10D). Scutellum with two flattened, semi-elliptical foveae on anterior margin (Plate 10D); with triangular, impunctate area antero-medially (Plate 10D); with numerous fine, short, longitudinal striae on posterior margin; with deep punctures, becoming larger towards alar foveae (Plate 10D); Mesopleuron without striae between punctures (Plate 10C). Metanotum gibbous, with large, deep, areolate-reticulate punctures (Plate 10D). Propodeum with lateral margin very slightly concave before propodeal angle; propodeal angle indistinct. Tarsal claw with three teeth.

*Metasoma*. Oval (Plate 10E), L/W = 15/11. T-I and T-II almost impunctate. T-III with fine, scattered punctures (Plate 10E); apex of T-III with narrow (1/3 MOD), colourless semi-transparent rim, without median notch (Plate 10F).

*Colouration*. Face metallic green. Head and mesosoma metallic blue, with purple reflections on vertex, median pronotum, and mesoscutum. Antenna black, with scape and pedicel metallic greenish-blue. Tegula metallic blue. Leg metallic greenish-blue, with tarsus brown. Metasoma purple, with metallic blue.

*Variation*. Female (n = 6). Body length 3.3–3.9 mm. Forewing length 2.6–3.2 mm.

*Male*. Chinese male specimens are not available for this study.

#### Distribution.

China (Yunnan), Laos, Russia, Turkey, Iran (Kimsey and Bohart 1991; Rosa et al. 2013).

#### Biology.

Unknown. Collected in March and July.

### Omalus potanini

(Semenov-Tian-Shanskij, 1932)

http://species-id.net/wiki/Omalus_potanini

Ellampus potanini Semenov-Tian-Shanskij 1932: 11.
Philoctetes (Holophris) potanini (Semenov-Tian-Shanskij, 1932): Tsuneki 1953: 55.
Omalus potanini (Semenov-Tian-Shanskij, 1932): Kimsey 1986: 107 (♂ lectotype designation); Kimsey and Bohart 1991: 249.

#### Material examined.

No material available for this study.

#### Diagnosis.

Body fully green-bronzy, with scutellum, metanotum, mesopleuron and lateral sides of the metasoma more or less golden-green. Tegula black, with faint metallic blue reflections. Mesoscutum smooth, polished, with notauli indistinct. Scutellum without flattened fovea on anterior margin; with broad, impunctate median area. Tarsal claw with three teeth. Metasoma oval. Apex of T-III with faint median notch (Semenov-Tian-Shanskij 1932).

#### Distribution.

China (Liaoning, Sichuan) (Semenov-Tian-Shanskij 1932; Tsuneki 1953; Kimsey and Bohart 1991).

#### Biology.

Unknown. Collected in July and August (Semenov-Tian-Shanskij 1932; Kimsey and Bohart 1991).

### Omalus probiaccinctus sp. n.

http://zoobank.org/92EB6218-3545-4859-85C5-3F4DC7ED9B3C

http://species-id.net/wiki/Omalus_probiaccinctus

Plates 11 , 12

#### Material examined.

Holotype: ♀ (SCAU), Guizhou, Suiyang, Kuankuoshui National Nature Reserve (27°55'24"N, 107°11'8"E), 4.VI.2010, Jie Zeng, No. SCAU-O0039. Paratype: 1 ♀ (SCAU), Guizhou, Suiyang, Kuankuoshui National Nature Reserve, 4.VI.2010, Jie Zeng, No. SCAU-O0040.

#### Diagnosis.

*Omalus probiaccinctus* sp. n. is related to *Omalus biaccinctus* based on the similar and peculiar punctures on the mesoscutum, which is unique in the West Palaearctic species. However, *Omalus probiaccinctus* sp. n. can be separated from the latter by having the body mostly metallic greenish-blue (body dark metallic blue or green, black medially on mesoscutum, metanotum, propodeum and metasoma in *Omalus biaccinctus*); tarsal claw with four teeth (three in *Omalus biaccinctus*); apex of T-III with faint median notch (apex of T-III with distinct, deep median notch in *Omalus biaccinctus*).

#### Description.

**Plate 11.**
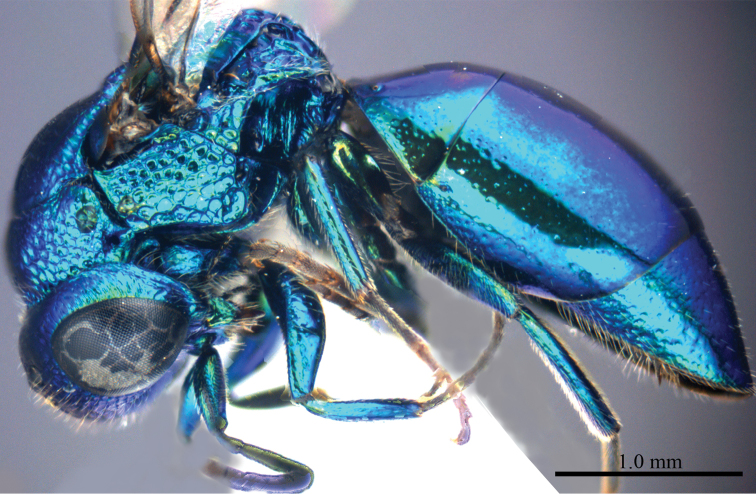
*Omalus probiaccinctus* sp. n., holotype, female. Habitus lateral.

*Female* (n = 2). Body length 4.8–5.7 mm (Plate 11). Fore wing length 4.1–4.4 mm. MS = 1.1 MOD.

**Plate 12.**
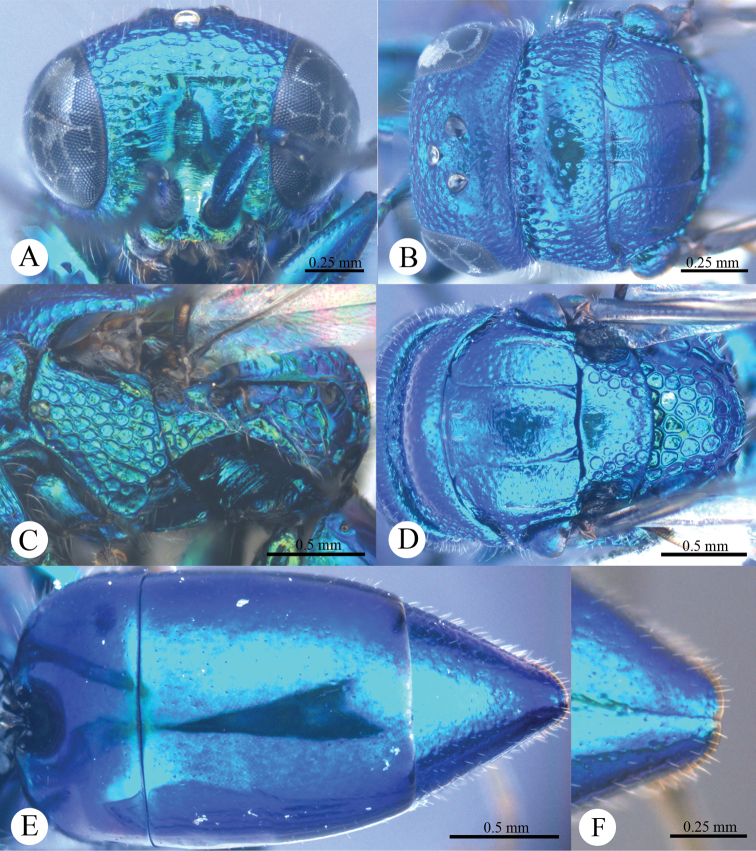
*Omalus probiaccinctus* sp. n., holotype, female. **A** Head anterior **B** Head and pronotum dorsal **C** Mesopleuron and metapleuron lateral **D** Mesoscutum, scutellum, metanotum and propodeum dorsal **E** Metasoma dorsal **F** Apex of T-III dorsal.

*Head*. Face with large round, dense (0–0.5 PD), and shallow punctures (Plate 12A). Scapal basin deep, glabrous, with weak and transverse striae (Plate 12A). Ocellar triangle isosceles. Postocellar line absent (Plate 12B). Gena with fine, oblique wrinkles.

*Mesosoma*. Pronotum with fine, scattered (> 2 PD) punctures medially; with small, deep pits on anterior margin; with large and dense (0–0.5 PD) punctures laterally (Plate 12B). Mesoscutum transversally rugulose, with fine, sparse punctures evenly scattered (Plate 12D); notauli distinct, deep, complete; notaulic pit elongate; parapsidal lines distinct, shallower than notauli (Plate 12D). Scutellum without flattened fovea on anterior margin; with broad, almost impunctate median area extend along its length, slightly convergent posteriorly (Plate 12D); posterior margin of scutellum with numerous fine, short, longitudinal striae; with large, round, dense (0–0.5 PD) punctures laterally, becoming larger, deeper towards alar foveae (Plate 12D). Mesopleuron with very weak striae between punctures (Plate 12C). Metanotum evenly round, with large, deep, areolate-reticulate punctures (Plate 12D). Propodeum with lateral margin concave before propodeal angle; propodeal angle distinct, pointing posterolaterally (Plate 12D). Tarsal claw with four teeth.

*Metasoma*. Distinctly elongate, L/W = 2/1, with T-III slightly constrict laterally towards the apex (Plate 12E). T-I and T-II almost impunctate dorsally, with fine, scattered punctures laterally. T-III with slightly denser punctures than those on T-II (Plate 12E); apex of T-III with narrow (1/5 MOD), brownish rim, with faint median notch (Plate 12F).

*Colouration*. Head and mesosoma metallic greenish-blue, with purple reflections. Antenna black, with scape and pedicel metallic green. Tegula metallic greenish-blue, with apex brown. Leg metallic greenish-blue, with tarsus brown. Metasoma metallic greenish-blue, with purple reflections.

*Male*. Unknown.

#### Distribution.

China (Guizhou).

#### Biology.

Unknown. Collected in June.

#### Etymology.

The name *probiaccinctus* is derived from the Latin preposition *pro*- and the chrysidid name *biaccinctus*.

### Omalus pseudoimbecillus sp. n.

http://zoobank.org/A2DABE4B-2BEF-4E28-8DD1-29D9D911A910

http://species-id.net/wiki/Omalus_pseudoimbecillus

Plates 13 , 14

#### Material examined.

Holotype: ♀ (SCAU), Yunnan, Yimen, Longquan Park (24°40'5"N, 102°9'2"E), 12.II.2005, He-sheng Wang, No. SCAU-O0041. Paratypes: 1 ♀ (SCAU), Yunnan, Yunlong, Tianchi National Nature Reserve (25°52'4"N, 99°17'25"E), 21.VIII.2003, Peng Wang, No. SCAU-O0042; 1 ♀ (SCAU), Yunnan, Jingdong, Jingping (24°27'14"N, 100°50'4"E), 28.IV.2005, He-sheng Wang, No. SCAU-O0043.

#### Diagnosis.

*Omalus pseudoimbecillus* sp. n. is similar to *Omalus imbecillus* based on the colouration, polished mesoscutum, and oval metasoma. However, it can be distinguished from the latter by having the tegula transparent brownish, with faint metallic reflections anteriorly (fully metallic blue in *Omalus imbecillus*); mesopleuron with striae between punctures (without striae in *Omalus imbecillus*); propodeal angle distinct and stout (indistinct in *Omalus imbecillus*).

#### Description.

**Plate 13.**
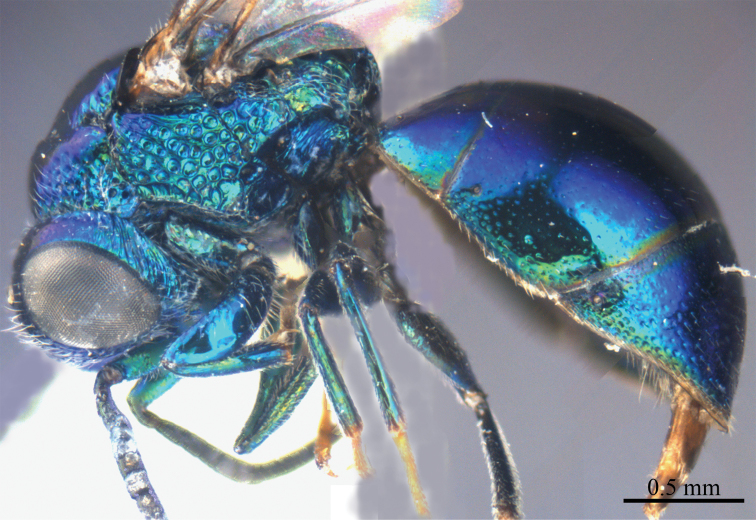
*Omalus pseudoimbecillus* sp. n., holotype, female. Habitus lateral.

*Female* (n = 3). Body length 3.9–4.4 mm (Plate 13). Fore wing length 3.3–3.6 mm. MS = 1.4 MOD.

**Plate 14.**
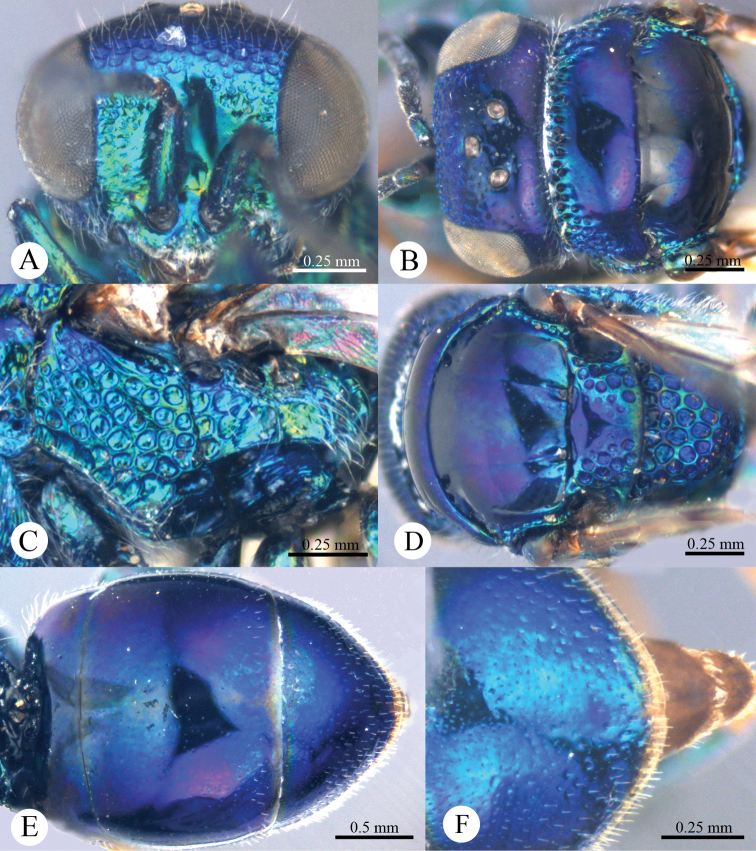
*Omalus pseudoimbecillus* sp. n., holotype, female. **A** Head anterior **B** Head and pronotum dorsal **C** Mesopleuron and metapleuron lateral **D** Mesoscutum, scutellum, metanotum and propodeum dorsal **E** Metasoma dorsal **F** Apex of T-III dorsal.

*Head*. Face with large, round, dense (0–0.5 PD), shallow punctures (Plate 14A). Scapal basin deep, smooth, glabrous (Plate 14A). Ocellar triangle isosceles. Postocellar line absent (Plate 14B). Gena with oblique wrinkles.

*Mesosoma*. Pronotum almost impunctate medially, with small, deep pits on anterior margin; with large, dense (0–0.5 PD) punctures laterally (Plate 14B). Mesoscutum polished, almost impunctate (Plate 14D); notauli indistinct but complete, impressed as fine lines, with notaulic pit round and short; parapsidal lines indistinct (Plate 14D). Scutellum with two flattened, semi-elliptical foveae on anterior margin (Plate 14D); with broad, impunctate median area extending along its length (Plate 14D); areolate-punctate laterally, becoming deeper, larger towards alar foveae (Plate 14D). Mesopleuron with striae between punctures (Plate 14C). Metanotum gibbous, with large, deep, areolate-reticulate punctate (Plate 14D). Propodeum with lateral margin concave before propodeal angle; propodeal angle distinct, stout, pointing posterolaterally. Tarsal claw with three teeth.

*Metasoma*. Oval (Plate 14E), L/W = 15/11. T-I and T-II almost impunctate dorsally, with fine, scattered punctures laterally. T-III with fine, denser punctures than those on T-II (Plate 14E); apex of T-III with narrow (1/3 MOD), testaceous semi-transparent rim, without median notch (Plate 14F).

*Colouration*. Face metallic blue. Vertex and mesosoma purple, with mesoscutum blackish, and mesopleuron metallic bluish-green or green. Antenna black, with scape and pedicel metallic green. Tegula transparent brownish, with faint metallic reflections anteriorly. Leg metallic greenish-blue, with tarsus testaceous. Metasoma blackish-purple, with metallic blue reflections.

*Male*. Unknown.

#### Distribution.

China (Yunnan).

#### Biology.

Unknown. Collected in February, April, and August.

#### Etymology.

The name *pseudoimbecillus* is derived from the Greek word psèydos (false) and the chrysidid name *imbecillus*. This name points to the morphological similarity between the two species.

### Omalus tibetanus sp. n.

http://zoobank.org/2DAB6611-C754-4092-8976-62D51990A738

http://species-id.net/wiki/Omalus_tibetanus

Plates 15 , 16

#### Material examined.

Holotype: ♀ (SCAU), Tibet, Chayu, Cibagou (28°55'0.59"N, 97°27'2.22"E), 3200 m, 22.VI.2009, Jiang-li Tan, No. 200902083.

#### Diagnosis.

*Omalus tibetanus* sp. n. is related to *Omalus probiaccinctus* sp. n. based on the distinctly elongate metasoma; to *Omalus berezovskii* Semenov-Tian-Shanskij based on the deep notauli. However, *Omalus tibetanus* sp. n. can be distinguished from them by the combination of the following characters: mesoscutum polished (transversally rugulose in *Omalus probiaccinctus* sp. n.); T-III median notch shallowly indented (median notch deeply V-shaped in *Omalus berezovskii*, slightly in *Omalus probiaccinctus* sp. n.); metasoma distinctly elongate (pear-shaped in *Omalus berezovskii*).

#### Description.

**Plate 15.**
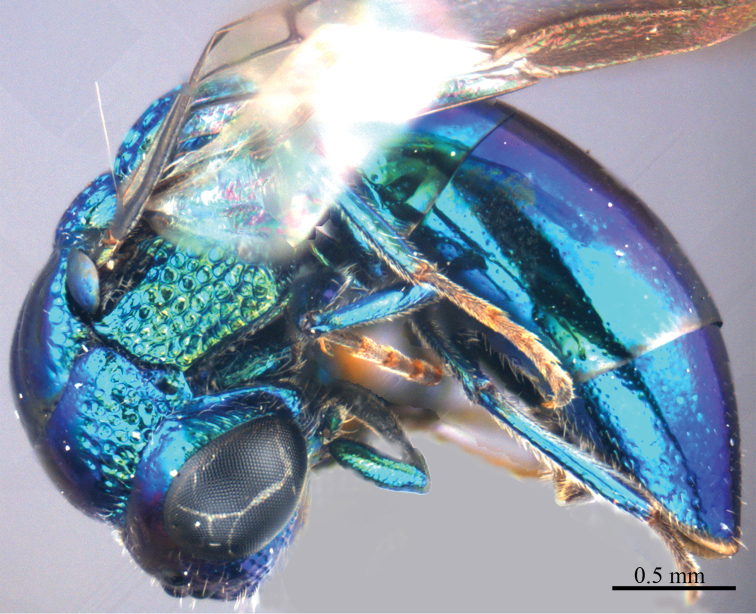
*Omalus tibetanus* sp. n., holotype, female. Habitus lateral.

*Female*. Body length 4.5 mm (Plate 15). Forewing length 3.9 mm. POL: OOL: OCL = 2.0: 2.7: 2.5. MS = 0.9 MOD.

**Plate 16.**
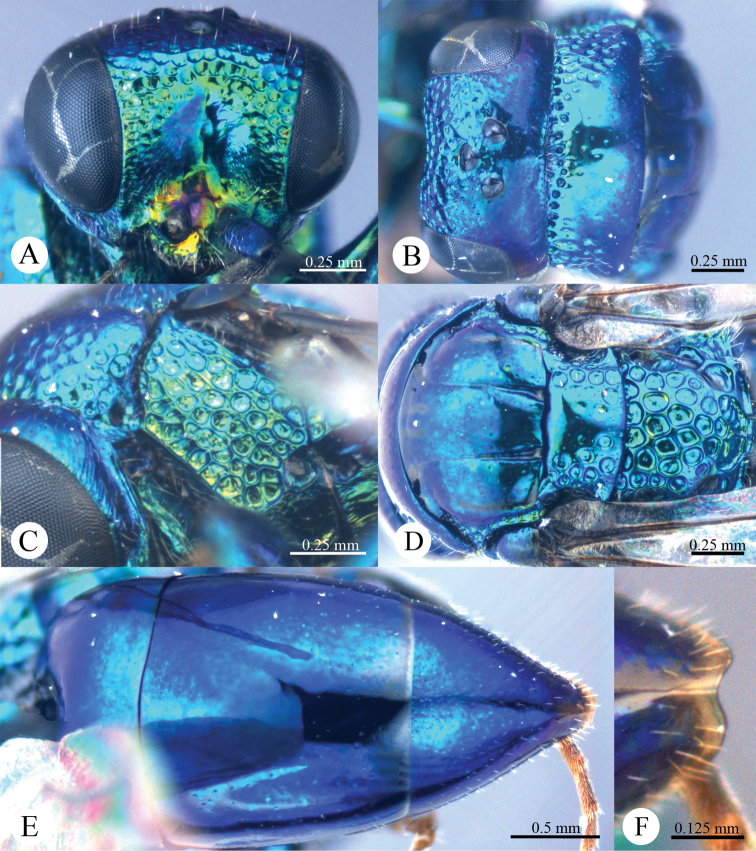
*Omalus tibetanus* sp. n., holotype, female. **A** Head anterior **B** Head and pronotum dorsal **C** Mesopleuron and metapleuron lateral **D** Mesoscutum, scutellum, metanotum and propodeum dorsal **E** Metasoma dorsal **F** Apex of T-III dorsal.

*Head*. Face with large, round, dense (0–0.5 PD), shallow punctures (Plate 16A). Scapal basin deep, with upper half smooth, glabrous; lower half weakly and obliquely striae laterally (Plate 16A). Ocellar triangle isosceles. Postocellar line absent (Plate 16B). Gena with oblique wrinkles.

*Mesosoma*. Pronotum almost impunctate medially (Plate 16B); with small, deep pits on anterior margin; with large, dense (0–0.5 PD) punctures laterally (Plate 16B). Mesoscutum polished, almost impunctate (Plate 16D); notauli distinct, deep, complete; notaulic pit elongate; parapsidal line shallower than notauli (Plate 16D). Scutellum without flattened fovea on anterior margin; with broad, impunctate median area extending along its full length (Plate 16D); shallowly areolate punctate laterally, becoming deeper, larger towards alar foveae (Plate 16D). Mesopleuron without striae between punctures (Plate 16C). Metanotum evenly round, large, deep, areolate-punctate (Plate 16D). Propodeum with lateral margin concave before propodeal angle; propodeal angle distinct, long, pointing posterolaterally (Plate 16D). Tarsal claw with three teeth.

*Metasoma*. Distinctly elongate, L/W = 13/9, with T-III slightly constrict laterally towards the apex (Plate 16E). T-I and T-II almost impunctate. T-III with fine, scattered punctures (Plate 16E); apex of T-III with narrow (1/2 MOD), testaceous transparent rim, with median notch shallowly indented (Plate 16F).

*Colouration*. Face metallic green, with some yellowish and violet tints on lower face between antennal socket. Vertex metallic blue, with purple laterally near the eye. Antenna black, with scape and pedicel metallic green. Mesosoma metallic bluish-green. Tegula metallic bluish-green, with apex blackish-brown. Leg metallic greenish-blue, with tarsus brown. Metasoma metallic blue, with purple reflections.

*Male*. Unknown.

#### Distribution.

China (Tibet).

#### Biology.

Unknown. Collected in June.

#### Etymology.

The species is named after the type locality.

## Supplementary Material

XML Treatment for Omalus

XML Treatment for Omalus aeneus

XML Treatment for Omalus berezovskii

XML Treatment for Omalus helanshanus

XML Treatment for Omalus imbecillus

XML Treatment for Omalus potanini

XML Treatment for Omalus probiaccinctus

XML Treatment for Omalus pseudoimbecillus

XML Treatment for Omalus tibetanus
